# Interface Microstructure and Properties of 42CrMo/Cr5 Vacuum Billet Forged Composite Roll

**DOI:** 10.3390/ma18010122

**Published:** 2024-12-31

**Authors:** Ming Li, Zongan Luo, Hongyu Zhou, Jingsong Yang, Guangming Xie, Guodong Wang, Jikui Liu, Weiguo Han, Shengpeng Xin

**Affiliations:** 1 State Key Laboratory of Rolling and Automation, Northeastern University, No. 3 Wenhua Road, Shenyang 110819, China; 1710219@stu.neu.edu.cn (M.L.);; 2 State Key Laboratory of Roll Composite Materials, Sinosteel Xing Tai Mechanical Roll Co., Ltd., No. 1 Xinxing West Street, Xingtai 054000, China

**Keywords:** composite roll, vacuum billet, interface microstructure, forging process, numerical simulation

## Abstract

Composite roll produced through casting methods typically remain in the as-cast state after forming. During the preparation process, extended exposure to high temperatures often results in microstructural coarsening at the interface and surface layers, restricting their mechanical performance. To overcome this limitation, we developed a novel vacuum billet forging process for the fabrication of composite rolls. By integrating numerical simulations with experimental validation, we successfully prepared a 42CrMo/Cr5 composite roll. The comprehensive characterization of the interface, including microstructure, elemental distribution, grain texture, grain type, and mechanical properties, was conducted using OM, SEM, EPMA, EBSD, Vickers hardness testing, and a universal testing machine. The relationship between the interface microstructure and mechanical performance was systematically analyzed. The results indicate that complete metallurgical bonding at the interface was achieved with an upsetting reduction ratio of 40% and a single-pass elongation reduction ratio of less than 10%. The interfacial microstructure consisted of four zones: the roll core exhibited lamellar pearlite and blocky ferrite; the diffusion layer near 42CrMo featured pearlite; the diffusion layer near Cr5 contained pearlite and Cr carbides; the Cr5 layer contained fine lamellar pearlite with a greater amount of dispersed Cr carbides. Significant diffusion of Cr and Ni elements was observed, with Cr diffusion extending to 70–90 μm. The interface grains experienced substantial deformation and recrystallization, enhancing the bonding strength. Tensile tests indicated that fracture occurred on the 42CrMo side, with yield and tensile strengths of 371 MPa and 729 MPa, respectively. The microhardness of the composite interface gradually increased from 190 HV to 305 HV without abrupt changes. A significant hardness difference was observed on both sides of the interface, while the variation within the diffusion layer was relatively smooth, indicating good bonding performance at the composite interface.

## 1. Introduction

Rolls are among the most critical consumables in steel rolling production, representing approximately 5% to 15% of total production costs [1,2]. Roll quality not only influences production expenses and mill operational efficiency but also directly affects the quality of rolled products [3,4,5,6,7,8]. During operation, rolls endure dynamic and static loads, wear, and thermal fluctuations. Thus, they must exhibit both high toughness to resist impact loads and sufficient hardness and wear resistance to minimize consumption [9,10]. However, traditional single-material rolls face limitations due to material properties, making it difficult to simultaneously achieve optimal core toughness and surface hardness/wear resistance [11,12]. To prevent roll breakage, more ductile but less wear-resistant materials are often used in production, resulting in faster wear, frequent replacements, and decreased production efficiency and economic viability. In contrast, composite rolls, which combine different materials in the core and outer layers, meet the dual demands for high surface wear resistance and core toughness, significantly extending service life [13,14]. This makes composite rolls a promising direction for future roll development.

Currently, the manufacturing processes for composite rolls are predominantly casting-based, including centrifugal casting [15,16,17,18], continuous casting [19,20,21], and electroslag remelting [22,23,24], each offering distinct advantages but also presenting inherent limitations. Centrifugal casting offers high production efficiency and cost-effectiveness but is hindered by poor interface bonding quality due to segregation and microdefects formed during high-speed solidification. Additionally, prolonged exposure to high temperatures promotes grain coarsening, which compromises the mechanical properties of the rolls. Continuous casting mitigates segregation issues and achieves improved interface bonding; however, it is characterized by high process complexity, stringent parameter control requirements, and low production efficiency. Extended high-temperature treatment further exacerbates grain coarsening at the interface, diminishing fatigue resistance under cyclic thermal loads. ESR, on the other hand, enhances interface purity and bonding strength through slag-layer purification. Despite these advantages, its high energy consumption and prolonged production cycles limit scalability, while local grain coarsening in the molten pool remains a significant challenge, restricting its suitability for high-performance composite roll production. Therefore, in casting-based composite roll production, the long residence time at high temperatures during the preparation process can cause grain coarsening at the interface and surface, limiting the mechanical properties of the rolls. In contrast, the forging process, through the application of external force and high-temperature plastic deformation, refines and densifies the grains of the roll material. Additionally, forging improves the uniformity of the internal microstructure, enhancing the consistency of its strength, toughness, and hardness [25]. However, there is limited research on the forging process for composite roll fabrication.

To address the limitations of traditional casting-based composite roll manufacturing processes, this study introduces an innovative vacuum billet forging process. By integrating vacuum electron beam welding with high-temperature forging, the process achieves complete metallurgical bonding at the interface. It ensures robust interface bonding, refines the microstructure, and offers key advantages such as high efficiency, low energy consumption, and environmental sustainability. This novel approach provides an advanced solution for manufacturing high-performance composite rolls while driving progress in composite material technology.

## 2. Materials and Methods

### 2.1. Materials

**Figure 1:**
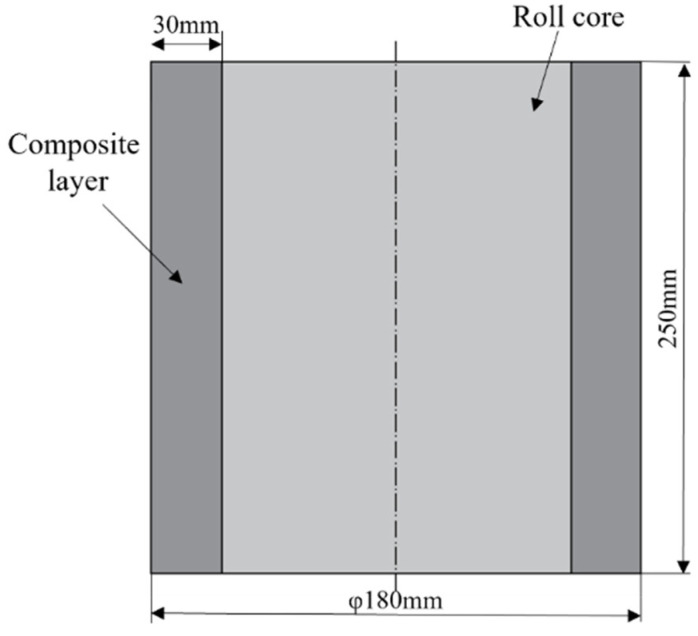
Longitudinal profile of roll core and composite layer.

It is well established that superior core toughness, along with high surface hardness and wear resistance, are critical attributes for high-performance rolls. In this study, medium-carbon alloy steel 42CrMo was selected as the core material, owing to its excellent toughness, forging formability, heat treatment response, and machinability [26]. The addition of Cr, Ni, and Mo further enhances its strength, making it well-suited for withstanding high stresses. For the composite layer, Cr5 was chosen due to its exceptional hardness and wear resistance [27,28]. This alloy is specifically alloyed with a high chromium content to provide superior wear performance, while the presence of V and Mo contributes to carbide precipitation, further improving its wear resistance. The detailed chemical compositions of 42CrMo and Cr5 are provided in Table 1, while the dimensions of the core and composite layer are depicted in Figure 1. The steel compositions conform to the following standards: 42CrMo steel is in accordance with GB/T 3077 [29], while Cr5 steel follows the standards of GB/T 1299 [30].

### 2.2. Simulation and Experimental Process

#### 2.2.1. Establishment of Finite Element Model

**Figure 2:**
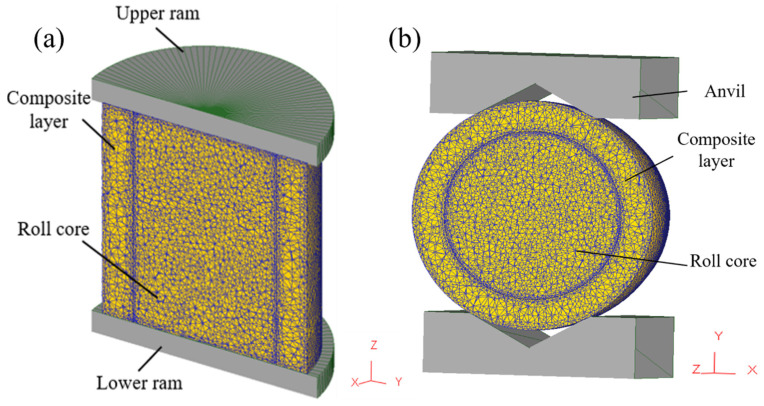
Three-dimensional thermal coupling model for composite roll: (**a**) upsetting; (**b**) elongation.

To improve simulation efficiency and reduce computation time, a 1/2 simulation model was used due to the symmetry of the forging process. In the simulation, the press head was treated as a rigid body, neglecting any elastic deformation. Both the core and composite layer were assumed to exhibit isotropic elastic–plastic behavior. JMatPro software(7.0) was used to obtain the temperature-dependent thermal properties of 42CrMo and Cr5, as detailed in Table 2 and Table 3. The three-dimensional thermo-mechanical coupling model of the composite forging process is illustrated in Figure 2, with Figure 2a depicting the upsetting stage and Figure 2b representing the elongation stage. The dimensions of the core were φ120 × 250 mm, while those of the composite layer were inner diameter 120 × outer diameter 180 × height 250 mm. During the forging process, the initial temperature of the composite billet was set to 1150 °C, while the upper and lower press heads were maintained at 200 °C, operating at a pressing speed of 10 mm/s. Shear friction was defined at the interface between the press head and composite billet, with a friction coefficient of 0.3. The upsetting reduction ratio is 40%, and the elongation reduction ratio is 10%. Assumptions included no relative displacement or energy loss on the symmetrical plane of the press head and a heat transfer coefficient of 45 N/(s·mm·°C) between the press head and composite billet. The external surface of the composite billet exchanged heat with the environment via radiation and convection, though the overall heat exchange was relatively minimal, resulting in a heat transfer coefficient of 0.02 N/(s·mm·°C). Stress variations within both the core and composite layer materials were analyzed at the 1/8, 1/4, 3/8, and 1/2 positions of the composite interface during the upsetting and elongation stages to evaluate the stress distribution across different regions.

#### 2.2.2. Experimental Procedure

**Figure 3:**
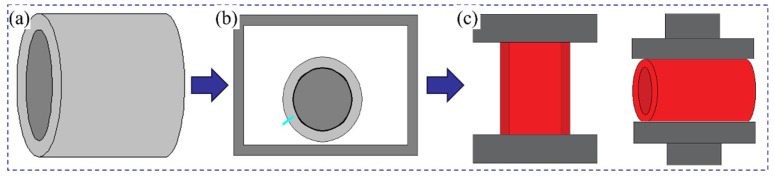
Vacuum billet forging composite process flowchart: (**a**) surface treatment and billet assembly; (**b**) vacuum electron beam welding for sealing; (**c**) high-temperature composite forging.

The fabrication of the 42CrMo/Cr5 composite roll involves vacuum electron beam welding for billet encapsulation, followed by high-temperature forging to achieve composite forming. The vacuum billet forging process consists of four critical steps: surface preparation, billet assembly, vacuum electron beam sealing, and high-temperature composite forging, as illustrated in Figure 3.

**Figure 4:**
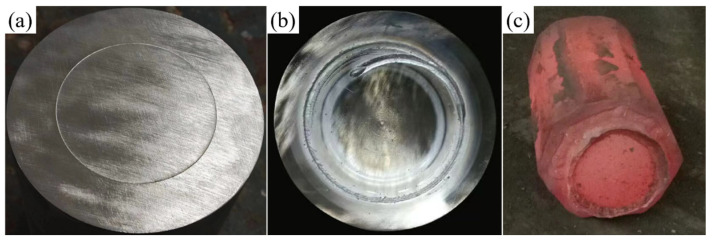
Experimental process illustrations: (**a**) schematic diagram after surface treatment and billet assembly; (**b**) schematic diagram after welding and sealing; (**c**) schematic diagram after forging and composite forming.

A clean interface between the core and composite layer was critical for achieving strong metallurgical bonding. During the preparation process, the machined billets were exposed to air, making the surfaces susceptible to oxidation, which can significantly weaken interface bonding strength. Thus, the removal of surface adsorption and oxide layers was essential. Initially, an electric grinder was employed to polish the surfaces of the core and composite layer to the specified dimensions, effectively removing iron oxide scales and inclusions. Subsequently, the polished surfaces were wiped with alcohol and acetone solutions to ensure the exposure of fresh, clean metal. Following surface treatment, the core was vertically inserted into the composite layer, with precise alignment maintained at both ends. Figure 4a provides an illustration of the surface treatment and billet assembly procedure.

The weld quality was crucial for the success of the subsequent high-temperature forging process. To prevent re-oxidation of the core and composite layer surface after cleaning, the billet assembly must be completed promptly and placed in the vacuum chamber of a vacuum electron beam welder for evacuation and sealing. Initially, the core and composite layer billets were securely clamped to ensure precise alignment at both ends. Circumferential seam welding was then performed to seal the composite billet. The welding parameters were set as follows: a welding voltage of 80 kV, a beam current of 60 mA, a welding speed of 300 mm/min, a vacuum pressure below 1 × 10^−2^ Pa, and the upper focus mode. Figure 4b presents the macrostructure of the vacuum electron beam-welded composite billet, showing no cracks or porosity, which confirms excellent weld quality.

The sealed composite billet was placed in a resistance furnace and gradually heated to 1150 °C, followed by a 2 h holding period to ensure uniform internal temperature distribution. Upsetting was then performed at a speed of 10 mm/s, maintaining a reduction rate of 40%. After the upsetting phase, V-anvil elongation was carried out with a single-pass reduction rate not exceeding 10% and three elongation passes, resulting in a final billet dimension of 172.4 × 273.1 mm. After elongation, the composite roll billet was placed in a 650 °C annealing furnace for prolonged heat preservation, followed by air cooling to room temperature. Figure 4c shows the composite roll billet immediately after elongation, where no weld seam cracking is observed.

### 2.3. Evaluation of Interface Structure and Mechanical Properties

**Figure 5:**
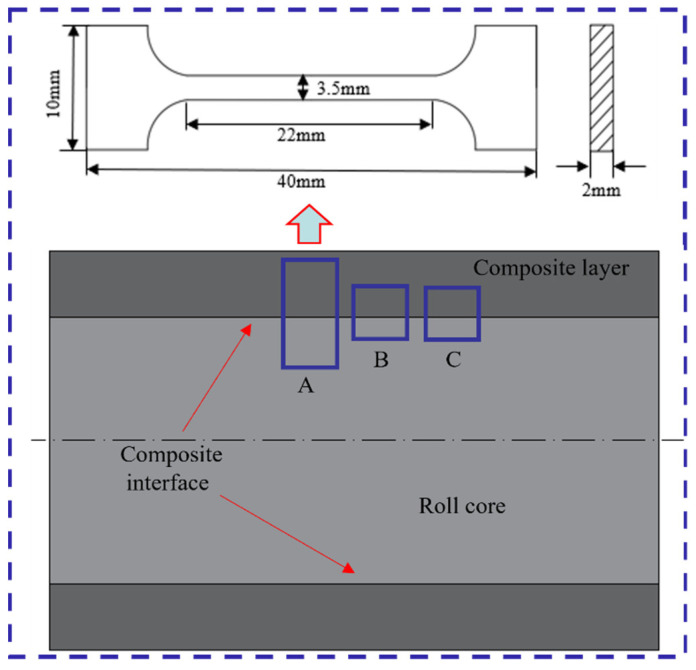
Schematic diagram of the analytical specimens.

As illustrated in Figure 5, the sampling locations for microstructure, tensile, and hardness specimens are designated as A, B, and C, respectively. The tensile specimen was extracted from position A, with specific dimensions indicated in the figure. Microstructure and hardness specimens were taken from positions B and C, each with dimensions of 10 × 10 × 10 mm. The specimens were etched using a 4% nitric acid alcohol solution, followed by characterization through optical microscopy (OM, Olympus, Tokyo, Japan), scanning electron microscopy (SEM, Thermo Fisher, Waltham, MA, USA), electron backscatter diffraction (EBSD, Oxford Instruments, Oxford, UK), and electron probe microanalysis (EPMA, JEOL, Tokyo, Japan) to evaluate the microstructure of the composite roll interface. Interface strength is a key metric for assessing the performance of composite rolls. To determine tensile strength, tensile specimens containing the interface were prepared and tested at room temperature using a universal testing machine (MTS Landmark, Eden Prairie, MN, USA), with a controlled tensile rate of 1 mm/min. Microhardness testing was conducted using an FM-810 microhardness tester (MHT, Future-tech, Fukui, Japan) with a 20 gf load, with multiple uniformly distributed indentations at the interface and on both sides, averaging the values to ensure the accuracy and reliability of the results.

## 3. Results and Analysis

### 3.1. Simulation of Forging Process for Composite Roll

**Figure 6:**
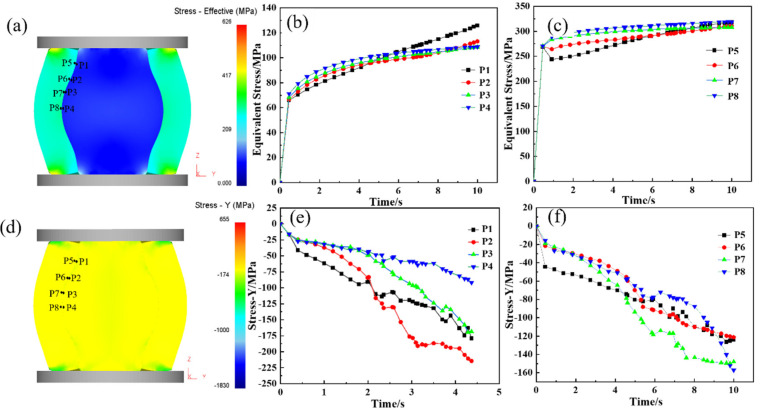
Stress analysis of the upsetting process of hot forging composite roll: (**a**) equivalent stress Diagram of composite roll with 50% upsetting reduction ratio; (**b**,**c**) equivalent stress curve of roll core and composite layer at the 1/8, 1/4, 3/8, and 1/2 upsetting positions of the composite interface; (**d**) stress-Y diagram of composite roll with 50% upsetting reduction ratio; (**e**,**f**) stress-Y curve of roll core and composite layer at the 1/8, 1/4, 3/8, and 1/2 positions of the composite interface.

During the upsetting process, the reduction rate is one of the main factors in determining the bonding quality at the core–composite interface. An insufficient reduction rate results in inadequate interface stress, preventing effective bonding, while an excessively high reduction rate, despite promoting good bonding, compromises elongation efficiency. Therefore, selecting an optimal reduction rate is crucial to achieving effective roll formation and enhanced elongation performance. Given the curved geometry of the composite interface, perpendicular forces are complex during upsetting. As a result, equivalent stress and Y-direction force are used as indicators for evaluating interface bonding. Figure 6 presents the equivalent stress distribution at the one-eighth, one-quarter, three-eights, and one-half positions along the interface during the hot forging of the composite roll. Figure 6a–c indicates that the equivalent stress at the core–composite interface increases with the reduction rate. When the reduction reaches 24.0% (at 6.0 s), the equivalent stress exceeds the deformation resistance, causing the interface to transition from the elastic to the plastic deformation stage. Figure 6d–f reveals that compressive stress develops at both the core and composite layer interfaces during upsetting, with the Y-directional pressure increasing as the reduction progresses, creating favorable conditions for metallurgical bonding. When the reduction reaches 40%, the Y-directional pressure surpasses the deformation resistance of both the core and composite layer, leading to significant plastic deformation and achieving strong bonding. During composite roll deformation, the core is constrained by the composite layer, resulting in the maximum stress occurring at the core–composite interface rather than at the core center. This promotes sufficient grain deformation at the interface, facilitating recrystallization.

**Figure 7:**
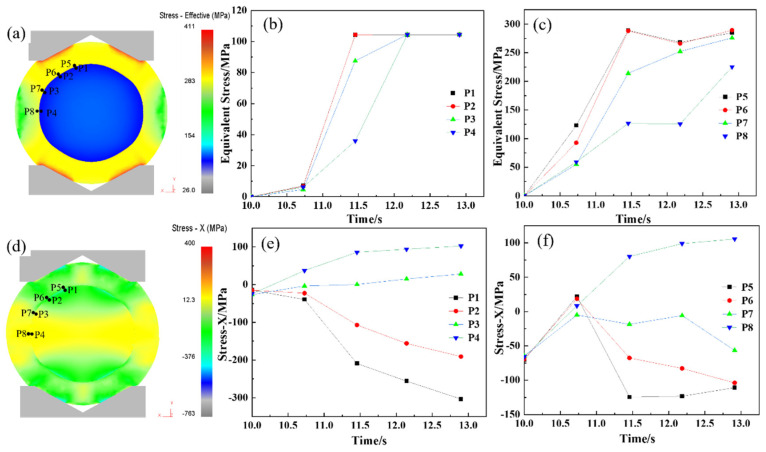
Stress analysis of elongation process of hot forging composite roll: (**a**) equivalent stress diagram of composite roll with 10% elongation reduction ratio; (**b**,**c**) equivalent stress curve of roll core and composite layer at the 1/8, 1/4, 3/8, and 1/2 positions of the composite interface; (**d**) stress-X diagram of composite roll with 10% elongation reduction ratio; (**e**,**f**) stress-X curve of roll core and composite layer at the 1/8, 1/4, 3/8, and 1/2 positions of the composite interface.

After the upsetting phase, complete metallurgical bonding was achieved between the core and composite layer. The elongation process was designed to achieve the final forged shape while maintaining the integrity of the interface, preventing any cracking. Figure 7 illustrates the distribution of equivalent stress and X-directional force at the interface during elongation. Figure 7a–c shows that equivalent stress at the interface increases progressively with the reduction. When the reduction reaches 9.0% (at 12.2 s), the equivalent stress surpasses the deformation resistance, marking the onset of plastic deformation at the interface. Figure 7d–f demonstrates that tensile stress develops along the X-direction during elongation, intensifying as the reduction proceeds. By 12.7 s, approximately 100 MPa of tensile stress forms at the interface, posing a risk to metallurgical bonding and potentially causing interface cracking. To mitigate this risk, a multi-pass elongation strategy with a small reduction per pass (10% reduction rate) was implemented, ensuring process stability and safety during forging.

### 3.2. Interface Microstructure Analysis of the Composite Roll

**Figure 8:**
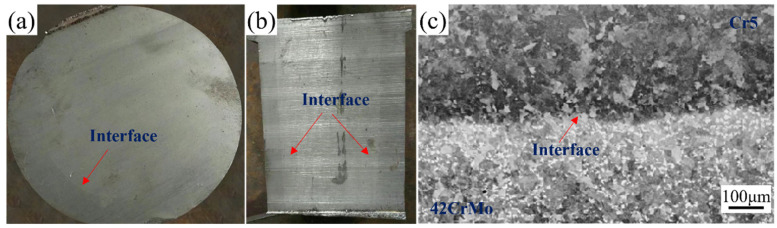
Macroscopic and microscopic images of the composite roll interface: (**a**) transverse cross-section; (**b**) longitudinal cross-section; (**c**) interface microstructure.

The bonding rate is a key parameter for assessing the performance of composite rolls. Comprehensive ultrasonic testing of the forged and annealed composite roll confirmed a 100% bonding rate. Figure 8a,b illustrates the transverse and longitudinal sections of the composite roll, both of which display a well-defined macroscopic bonding interface. Figure 8c presents the metallographic image of the interface, showing effective bonding with no unbonded regions or microcracks, indicating complete metallurgical integration between the core and composite layer.

**Figure 9:**
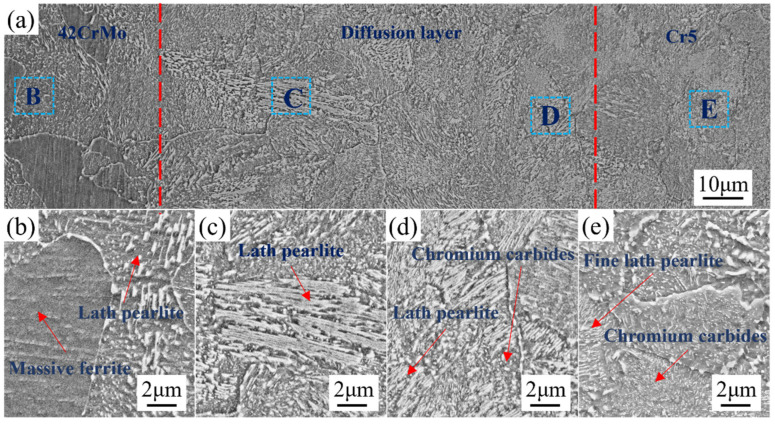
Microstructure at the interface of the composite roll: microstructures of (**a**) composite interface; (**b**) 42CrMo; (**c**,**d**) intermediate diffusion layer; (**e**) Cr5.

Figure 9 presents the interface scanning image of 42CrMo/Cr5. Figure 9a highlights the composite interface, with the 42CrMo roll core located on the left and the Cr5 composite layer on the right. The diffusion layer, delineated by the red dashed region, measures approximately 60–80 μm in width. As shown, high-temperature plastic deformation facilitated extensive dynamic recrystallization in both the 42CrMo roll core and the Cr5 composite layer, resulting in a transitional microstructure at the interface without distinct boundaries. This observation confirms the achievement of complete metallurgical bonding at the roll interface, ensuring the continuity of elements and microstructures through the combined effects of high-temperature forging and diffusion processes. Dynamic recrystallization is crucial in the forging process, particularly in preventing crack propagation. It refines the grain structure, enhancing plasticity and toughness, thereby reducing stress concentration and crack propagation risk. Additionally, it alleviates internal stresses accumulated during forging, which are potential sources of cracks. By rearranging the lattice structure, dynamic recrystallization effectively reduces these internal stresses, lowering the likelihood of crack extension. In composite material forging, it also strengthens interphase bonding, preventing interfacial delamination or cracking. Finally, dynamic recrystallization improves thermal stability, further reducing the risk of crack propagation under high temperatures [31]. Figure 9b depicts the microstructure at point B in Figure 9a, corresponding to the 42CrMo roll core. The microstructure is primarily characterized by blocky ferrite and lamellar pearlite. This structure forms as a result of the immediate transfer of the forged composite billet into a high-temperature furnace maintained at 680 °C for soaking. Under these conditions, 42CrMo undergoes a full phase transformation. The blocky ferrite represents the proeutectoid phase, while the lamellar pearlite is a product of the eutectoid reaction. This microstructural arrangement imparts excellent plasticity and toughness to the 42CrMo material. Figure 9c displays the microstructure morphology of the diffusion layer near the 42CrMo roll core side, predominantly consisting of lamellar pearlite. The significant differences in Cr and Ni element concentrations between 42CrMo and Cr5 create a pronounced compositional gradient, driving element diffusion. During slow cooling, the elevated Cr content effectively suppresses the precipitation of proeutectoid ferrite, leading to the formation of a lamellar pearlite-dominated microstructure. Figure 9d illustrates the microstructure of the diffusion layer adjacent to the Cr5 composite layer, which primarily comprises lamellar pearlite and chromium carbides. Compared to the region near the 42CrMo side, the lamellar spacing of pearlite in this area is markedly reduced, accompanied by an increased density of chromium carbide precipitates. This refinement is attributed to the higher Cr content, which combines with carbon to form stable chromium carbides. Figure 9e demonstrates the microstructure of the Cr5 composite layer, featuring finer lamellar pearlite interspersed with numerous chromium carbides. The high Cr content in the Cr5 composite layer reacts with carbon to generate abundant chromium carbides. These carbides not only enhance the hardness of the structure but also act as effective pinning agents during the nucleation and growth of pearlite, leading to substantial refinement of the microstructure. The combination of refined lamellar pearlite and uniformly distributed chromium carbides imparts the Cr5 composite layer with exceptional hardness and superior wear resistance [32,33].

### 3.3. Elemental Analysis of the Interface in the Composite Roll

**Figure 10:**
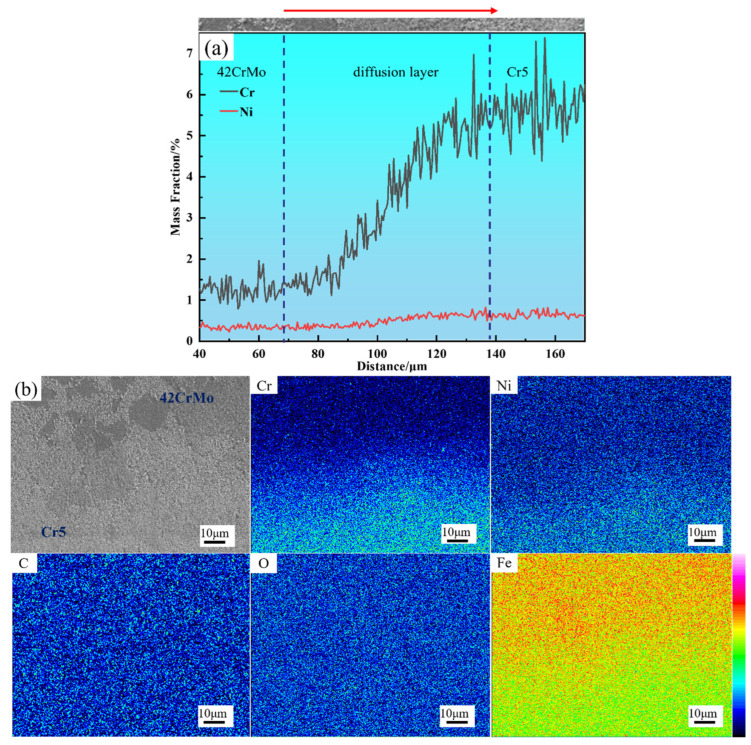
Element distribution of composite interface: (**a**) line scanning analysis of composite interface; (**b**) surface scanning analysis of composite interface.

Figure 10a presents the diffusion curves at the interface, showing that Cr and Ni diffuse toward the 42CrMo core, with Cr demonstrating a diffusion width of approximately 70–90 μm, which is slightly broader than the measured diffusion layer. This is attributed to the fact that microstructural changes are less discernible when elemental concentration differences are minimal. Figure 10b illustrates the distribution of elements across the interface between the composite layer and the core, revealing no significant accumulation of other elements at the bonding interface. While small amounts of oxides or carbides may remain on the surfaces of the core and composite layer post-treatment, their concentrations are minimal. During subsequent forging, these oxides and carbides are broken down and evenly dispersed along the interface, preventing notable accumulation. Furthermore, the composite billet’s welding process was conducted under a vacuum pressure of 10^−2^ Pa, effectively minimizing air intrusion at the metal contact surface. After welding, the composite billet interface was hermetically sealed, completely isolating it from external air. This explains the absence of significant carbon or oxygen accumulation at the interface.

### 3.4. EBSD Analysis of the Interface in the Composite Roll

**Figure 11:**
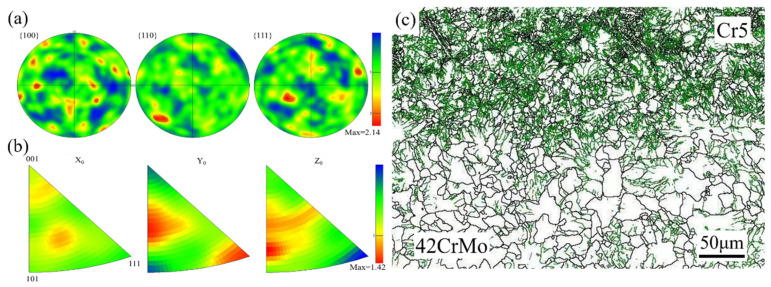
(**a**) Pole figure of the composite interface microstructure; (**b**) inverse pole figure of the composite interface microstructure; (**c**) orientation difference angle distribution at the composite interface.

Figure 11a presents the pole figure of the composite interface, showing no distinct strong texture formation. The pole density points exhibit a random distribution with dispersed texture, and the texture strength is relatively low at 2.14. Figure 11b depicts the inverse pole figure of the composite interface, indicating that most grains have a <111> pole axis aligned with the radial direction, a feature that contributes to enhanced interface strength [34]. Figure 11c reveals a high density of low-angle grain boundaries within the grains, indicating significant dislocation accumulation at the composite interface. During deformation, grain boundaries are prone to tilting and twisting, which impedes dislocation motion. As deformation progresses, dislocation density within the material increases, and dislocations intertwine to form dislocation walls. These walls eventually evolve into sub-grain boundaries, facilitating sub-grain structure development and dynamic recovery. Upon reaching a critical stress level, sub-grain boundaries continue to absorb dislocations, ultimately transforming into high-angle grain boundaries, signifying dynamic recrystallization. Consequently, deformed grains exhibit a high density of dislocations, which typically form deformation boundaries [35,36].

**Figure 12:**
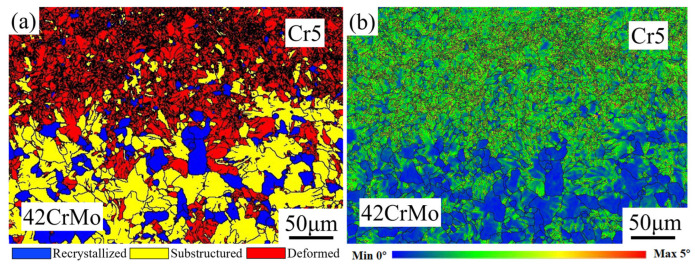
(**a**) EBSD micrograph of composite interface; (**b**) local orientation difference distribution map of the composite interface.

Figure 12a presents the EBSD grain-type map at the interface between the roll core and the composite roll. The composite layer (Cr5) of the hot-forged composite roll predominantly features deformed grains and recrystallized grains, with relatively few sub-grains. In contrast, the roll core side is primarily composed of sub-grains and recrystallized grains, along with a small fraction of deformed grains. Notably, the composite interface region is enriched with a substantial number of recrystallized grains. The formation of these recrystallized grains is mainly attributed to the significant plastic deformation experienced by the composite interface during the forging process, which induces dynamic recrystallization. This phenomenon markedly enhances the interface’s strength and bonding performance. During the deformation of metallic materials, inherent microstructural heterogeneities or externally applied non-uniform stress fields often lead to the development of plastic strain gradients. Geometrically necessary dislocations (GNDs), which are essential for accommodating non-uniform deformation, are typically concentrated along these strain gradients. In the composite roll, the interface region, due to its distinct processing pathway and stress state, tends to accumulate GNDs, thereby promoting recrystallization. This microstructural evolution not only strengthens the composite interface but also lays the groundwork for subsequent strengthening mechanisms. Figure 12b illustrates the local misorientation distribution map at the composite roll interface. The green regions in the map, indicative of a higher strain distribution, are predominantly located within deformed grains and sub-grains. These areas exhibit significant strain gradients, particularly at the boundaries between deformed grains and adjacent grains, where pronounced strain concentrations are observed. Such regions of strain concentration are potential sites for micro-crack initiation and propagation. However, they also act as powerful driving forces for dynamic recrystallization, facilitating further microstructural refinement.

### 3.5. Analysis of Mechanical Performance at the Composite Roll Interface

**Figure 13:**
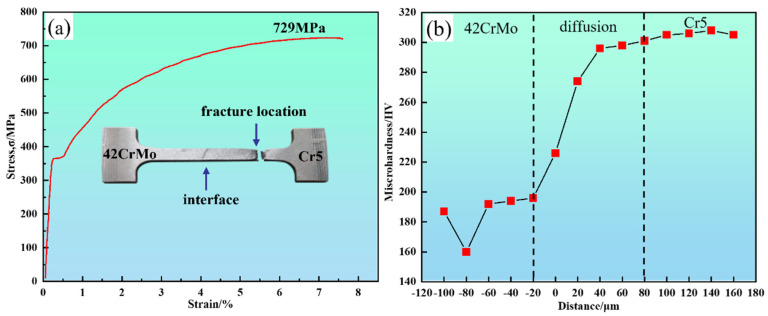
The mechanical properties of the 42CrMo/Cr5 composite roll interface: (**a**) tensile stress-strain curve of the composite interface; (**b**) hardness distribution of the composite interface.

A set of tensile tests was conducted, with representative results shown in Figure 13a, to evaluate the mechanical properties of the 42CrMo/Cr5 composite roll interface. The results, based on tensile testing and hardness measurements, indicate that the interface exhibits excellent bonding strength and a well-defined hardness gradient, as summarized in Figure 13 and Table 4. The tensile test results show that fracture occurred approximately 8–10 mm from the interface in the 42CrMo region, with a yield strength of 370 MPa, tensile strength of 729 MPa, and elongation of 7.7%. The visible interface at the center of the specimen confirms that it is not the weakest link, further demonstrating the robust bonding strength of the composite roll. The significant disparity in chromium content between 42CrMo and Cr5 led to chromium diffusion toward the 42CrMo side during high-temperature forging, resulting in solid solution strengthening. Additionally, dispersed carbides at the interface contributed to the strength of the bond [37]. Consequently, the 42CrMo region emerged as the weakest link, fracturing once the stress limit was reached.

The hardness distribution, presented in Figure 13b, was measured perpendicular to the interface at 20 μm intervals. The results reveal that the lowest hardness values occur on the 42CrMo side due to its microstructure of blocky ferrite (160 HV) and lamellar pearlite (190 HV). In contrast, the Cr5 side exhibits significantly higher hardness, reaching approximately 305 HV. Within the intermediate diffusion region, hardness values increase gradually from 190 HV to 305 HV without abrupt transitions. This continuous gradient is attributed to chromium diffusion, which enhances hardness through solid solution strengthening and second-phase strengthening mechanisms during the forging process.

The diffusion of chromium and nickel plays a critical role in enhancing the bond strength, hardness, and wear resistance of the interface in 45 steel and semi-high-speed steel composites. Chromium, with its relatively large diffusion width, promotes the formation of stable solid solution phases and chromium carbides with the base metal, significantly improving interface bond strength and hardness. This diffusion also enhances the chemical stability of the interface, improving wear resistance and reducing friction-induced damage [38]. In contrast, nickel, despite having a smaller diffusion width, primarily enhances the interface’s plasticity and toughness in localized regions. Nickel diffusion improves ductility, prevents crack propagation under thermal stress and friction, and helps alleviate stress concentration at the interface, thereby improving crack resistance and overall interface stability [39].

### 3.6. Comparison Between Vacuum Billet Forging and Traditional Casting-Based Composite Roll Manufacturing Processes and Future Development Directions

#### 3.6.1. Comparison Between Vacuum Billet Forging and Traditional Casting-Based Composite Roll Manufacturing Processes

To thoroughly assess the advantages of the vacuum billet forging process, a comparison with traditional casting methods, including centrifugal casting, continuous casting, and electroslag remelting, is conducted, focusing on performance, cost, scalability, and environmental impact, as shown in Table 5.

The vacuum billet forging process offers significant advantages over traditional casting methods in terms of interface quality, mechanical performance, and environmental impact, making it a preferred choice for producing high-performance composite rolls. While centrifugal casting remains the most cost-effective and scalable option for large-scale production, its relatively weaker interface bonding limits its application in demanding environments [40]. Continuous casting and electroslag remelting provide good performance but come with higher costs and lower scalability.

Compared to these traditional casting methods, the vacuum billet forging composite technology excels in reducing material segregation and improving hardness gradients. In conventional casting, uneven cooling rates often lead to the segregation of alloying elements, resulting in non-uniform microstructures and variations in performance. However, the vacuum billet forging process effectively mitigates this issue by controlling temperature and strain rates, reducing segregation, and promoting a more uniform distribution of elements throughout the material. This results in a more consistent microstructure, significantly enhancing the material’s mechanical properties, particularly its strength and toughness. Moreover, the vacuum billet forging process improves the hardness gradient, which is often problematic in traditional casting due to cooling rate differences across the material’s cross-section. With precise temperature control and high-temperature forging, the vacuum billet forging process ensures a more uniform hardness distribution. This optimized hardness gradient contributes to the long-term performance and reliability of composite materials, especially in harsh working conditions.

The vacuum billet forging composite technology proposed in this study, especially in the application of 45 steel and semi-high-speed steel composites, has significant practical implications. By adopting this manufacturing method, we can achieve uniform grain sizes in the interface and composite layer regions of the rolls, thereby improving the material’s microstructure. This significantly extends the service life of the rolls, reduces the frequency of replacements and maintenance, and lowers production costs.

Additionally, the diffusion of chromium and nickel plays an important role in optimizing the thermal stability and high-temperature performance of the rolls, especially in cold and hot working rolls operating at high temperatures. By enhancing the interface bonding strength, this method effectively reduces crack propagation under high-temperature conditions, improving the reliability and stability of the rolls in harsh working environments.

#### 3.6.2. Future Development Directions

This study provides valuable insights into the vacuum billet forging process, but it also has limitations. Experimental constraints prevented full control over certain variables, which may have influenced the results. Additionally, the focus on specific materials limits the generalizability of the findings. Future research should expand the experimental scope to include a wider range of materials and conditions, along with long-term performance evaluations. Advancing experimental techniques and developing new simulation models will be crucial for refining and validating the conclusions. With the widespread adoption of this technology, the roll manufacturing industry is expected to see significant improvements in quality, efficiency, and cost control. Looking forward, the vacuum billet forging process holds great potential for advancing composite roll manufacturing. Future developments may focus on optimizing equipment, reducing costs, and enhancing scalability.

## 4. Conclusions

(1) Under the conditions of heating to 1150 °C and holding for 2 h, complete metallurgical bonding at the composite roll interface is achieved with a 40% upsetting reduction rate and a single-pass elongation reduction not exceeding 10%. This process was successfully predicted through simulation and further validated experimentally.

(2) The interfacial microstructure consisted of four zones: the roll core exhibited lamellar pearlite and blocky ferrite; the diffusion layer near 42CrMo featured pearlite; the diffusion layer near Cr5 contained pearlite and Cr carbides; the Cr5 layer, composed of fine lamellar pearlite with a greater amount dispersed Cr carbides.

(3) The interface width measures approximately 60–80 μm, with no visible unbonded areas or oxide/carbide accumulation, indicating uniform bonding. Chromium diffusion at the interface spans 70–90 μm, while the grains at the interface exhibit significant deformation and recrystallization, which enhances interfacial strength.

(4) The yield strength of the composite interface reaches 371 MPa, while the tensile strength is measured at 729 MPa, with fracture occurring on the 42CrMo side. The microhardness of the composite interface gradually increases from 190 HV to 305 HV without abrupt transitions. A significant hardness gradient exists between the two sides of the interface, while changes within the diffusion layer are relatively smooth, indicating strong bonding performance at the composite interface.

## Figures and Tables

**Table 1:** Chemical composition mass fraction of roll core and composite layer (%).

Element	C	Si	Mn	Cr	Ni	Mo	V	Fe
Roll core	0.42	0.25	0.78	1.08	0.36	0.23	0.00	Bal.
Composite layer	0.45	0.35	0.43	4.85	0.72	0.25	0.12	Bal.

**Table 2:** Thermal and physical properties of 42CrMo.

Temperature (°C)	Elasticity Modulus (GPa)	Specific Heat Capacity [kJ/(kg·°C)]	Thermal Conductivity [N/(s·°C)]	Coefficient of Linear Expansion (1/°C)
950	114.16	0.62	28.02	2.27
1000	109.34	0.63	28.66	2.40
1050	103.76	0.64	29.25	2.52
1100	98.86	0.65	29.86	2.65
1150	93.92	0.66	30.45	2.77
1200	88.96	0.67	31.04	2.89

**Table 3:** Thermal and physical properties of Cr5.

**Temperature** **(°C)**	**Elasticity** **Modulus (GPa)**	**Specific Heat** **Capacity** **[kJ/(kg·°C)]**	**Thermal Conductivity** **[N/(s·** **°C)]**	**Coefficient of** **Linear Expansion (1/** **°C)**
950	116.08	0.76	27.80	2.25
1000	110.95	0.62	28.64	2.38
1050	106.93	0.63	29.22	2.51
1100	101.62	0.64	29.81	2.64
1150	96.91	0.65	30.39	2.76
1200	92.17	0.66	30.97	2.88

**Table 4:** The mechanical properties of the 42CrMo/Cr5 composite interface.

Yield Strength (MPa)	Tensile Strength (MPa)	Elongation (%)	Hardness Range (HV)
360~370	725~729	7.0~7.7%	190~305

**Table 5:** Comparison of composite roll manufacturing processes.

	Vacuum Billet Forging	Centrifugal Casting	Continuous Casting	Electroslag Remelting
Performance	Superior interface quality with refined microstructure	Moderate interface quality, prone to segregation and weaker bonding	Good bonding with reduced segregation	High bonding Quality, fine structure but energy-intensive
Cost	Higher initial cost due to specialized equipment	Low, cost-effective for mass production	Higher cost due to energy demand and longer processing	High cost, largely due to energy consumption
Scalability	Suitable for small- to large-scale production with high performances	Excellent for large-scale production	Moderate scalability, requires precise control	Limited Scalability, due to long processing time
Environmental Impact	Low emissions	Moderate emissions, molten metal handling contributes to oxidation	Moderate, requires significant energy	High energy consumption and environmental emissions

## Data Availability

The original contributions presented in the study are included in the article, and further inquiries can be directed to the corresponding author.
